# Tetramic Acids Mutanocyclin and Reutericyclin A, Produced by *Streptococcus mutans* Strain B04Sm5 Modulate the Ecology of an *in vitro* Oral Biofilm

**DOI:** 10.3389/froh.2021.796140

**Published:** 2022-01-07

**Authors:** Carla Uranga, Karen E. Nelson, Anna Edlund, Jonathon L. Baker

**Affiliations:** ^1^Genomic Medicine Group, J. Craig Venter Institute, La Jolla, CA, United States; ^2^Department of Pediatrics, UC San Diego School of Medicine, San Diego, CA, United States

**Keywords:** *Streptococcus mutans* (*S. mutans*), oral microbiota, biofilm, dental caries, tetramic acid, oral microbiome

## Abstract

The human oral microbiome consists of diverse microbes actively communicating and interacting through a variety of biochemical mechanisms. Dental caries is a major public health issue caused by fermentable carbohydrate consumption that leads to dysbiosis of the oral microbiome. *Streptococcus mutans* is a known major contributor to caries pathogenesis, due to its exceptional ability to form biofilms in the presence of sucrose, as well as to its acidophilic lifestyle. *S. mutans* can also kill competing bacteria, which are typically health associated, through the production of bacteriocins and other small molecules. A subset of *S. mutans* strains encode the *muc* biosynthetic gene cluster (BGC), which was recently shown to produce the tetramic acids, mutanocyclin and reutericyclins A, B, and C. Reutericyclin A displayed strong antimicrobial activity and mutanocyclin appeared to be anti-inflammatory; however the effect of these compounds, and the carriage of *muc* by *S. mutans*, on the ecology of the oral microbiota is not known, and was examined here using a previously developed *in vitro* biofilm model derived from human saliva. While reutericyclin significantly inhibited *in vitro* biofilm formation and acid production at sub-nanomolar concentrations, mutanocyclin did not present any activity until the high micromolar range. 16S rRNA gene sequencing revealed that reutericyclin drastically altered the biofilm community composition, while mutanocyclin showed a more specific effect, reducing the relative abundance of cariogenic *Limosilactobacillus fermentum*. Mutanocyclin or reutericyclin produced by the *S. mutans* strains amended to the community did not appear to affect the community in the same way as the purified compounds, although the results were somewhat confounded by the differing growth rates of the *S. mutans* strains. Regardless of the strain added, the addition of *S. mutans* to the *in vitro* community significantly increased the abundance of *S. mutans* and *Veillonella infantium*, only. Overall, this study illustrates that reutericyclin A and mutanocyclin do impact the ecology of a complex *in vitro* oral biofilm; however, further research is needed to determine the extent to which the production of these compounds affects the virulence of *S. mutans*.

## Introduction

The oral microbiota contains a metabolically dynamic community of bacteria, ranging from health-associated commensals, to opportunistic pathogens and overtly pathogenic taxa [1–3]. The majority of the signaling and socio-chemical relationships underpinning the ecology of this complex community are not well understood [4]. Caries (tooth decay), gingivitis (a mild form of gum disease), and periodontal disease (severe gum disease) represent some of the most common public health issues that are caused by dysbiosis (a disruption to microbiota homeostasis), in part as a result of host behaviors, such as diet and poor oral hygiene [5, 6]. A diet with frequent sugar intake not only provides abundant carbohydrate sources for biofilm-forming acidogenic (acid-producing) bacteria, but also results in a highly acidic environment that selects for the growth of aciduric (acid-tolerant) bacteria, followed by the demineralization (erosion) of tooth enamel and caries disease progression [1, 5, 6].

**Figure 1:**
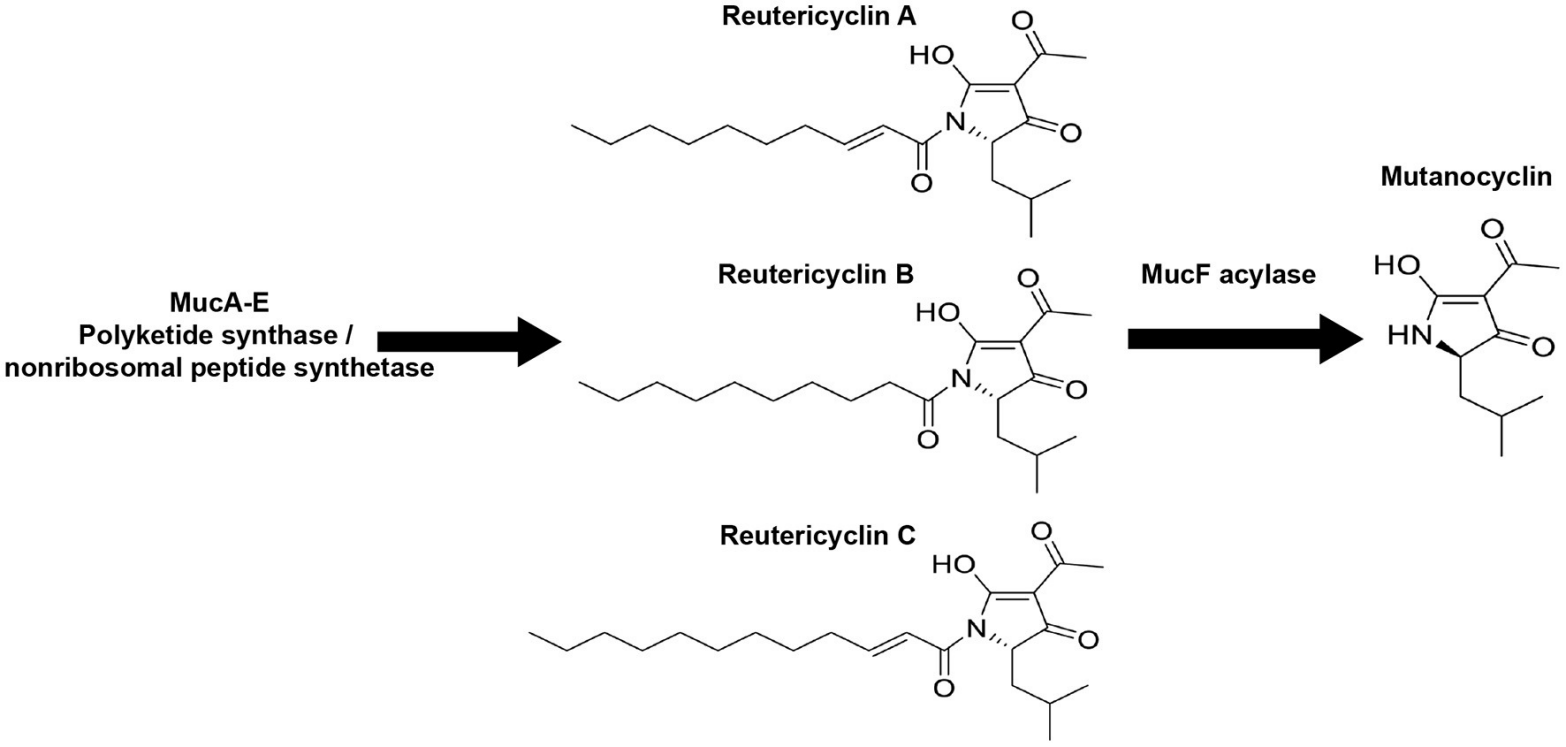
The *Streptococcus mutans* B04Sm5 *muc* biosynthetic gene cluster (BGC) produces reutericyclins **(A–C)** and mutanocyclin. Illustration showing the chemical structures of reutericyclins **(A–C)** and mutanocyclin. Reutericyclins are made by MucA-E and are then processed by the MucF acylase to yield mutanocyclin [16].

Since the turn of the 19th century, the studies of dental plaque (microbial biofilms growing on teeth) have recognized *Streptococcus mutans* as a major caries-associated pathogen, which is attributed to its capacity to produce copious amounts of lactic acid, while forming agglutinative biofilms on teeth [7, 8]. Recent findings reveal that *S. mutans* virulence traits, biofilm formation, acid production, and the biosynthesis and secretion of small molecules (e.g., bacteriocins, non-ribosomal peptides, and polyketides) vary widely between strains [9–12]. Previous studies identified that a subset of *S. mutans* strains (22 of the 169 strains examined by Liu et al.) encoded a biosynthetic gene cluster (BGC) that was highly homologous to the reutericyclin BGC, which was originally identified in the genomes of *Lactobacillus reuteri* [13–16]. The biosynthetic capacity of this new *muc* BGC was further characterized, resulting in the identification of four tetramic acid products; reutericyclins A, B, and C, and mutanocyclin (Figure 1) [16]. Mutanocyclin is the tetramic acid core common to all four compounds, while reutericyclins A-C have different fatty acid chains at the N-1 position of the tetramic acid core. Activity testing of the compounds revealed that reutericyclin A (the only reutericyclin tested here and referred to throughout this study as just “reutericyclin”) exerted antimicrobial activities against a broad range of Gram-positive bacteria [16–18]. A proton ionophore, reutericyclin, causes proton diffusion through the cell membranes of sensitive bacteria [17, 19], thus abolishing the pH differential across the membrane. Mutanocyclin, less well understood, was not reported to have any antimicrobial activity but showed anti-inflammatory responses in a murine model [15]. Although reutericyclin (produced by *S. mutans* B04Sm5) inhibited the growth of neighboring commensal streptococci, its role in a broader ecology of the complex oral microbiota was not known [16]. Furthermore, it is unclear if the carriage of *muc* affects *S. mutans* pathogenicity in caries-associated dysbiosis. In this study, the effects on oral biofilm growth, bacterial taxonomic diversity, and community gene transcription activities, were examined by the introduction of reutericyclin A, mutanocyclin, and *S. mutans* strains which produce one, both, or neither of these compounds into a complex *in vitro* biofilm model derived from human saliva [20, 21].

## Results

**Figure 2:**
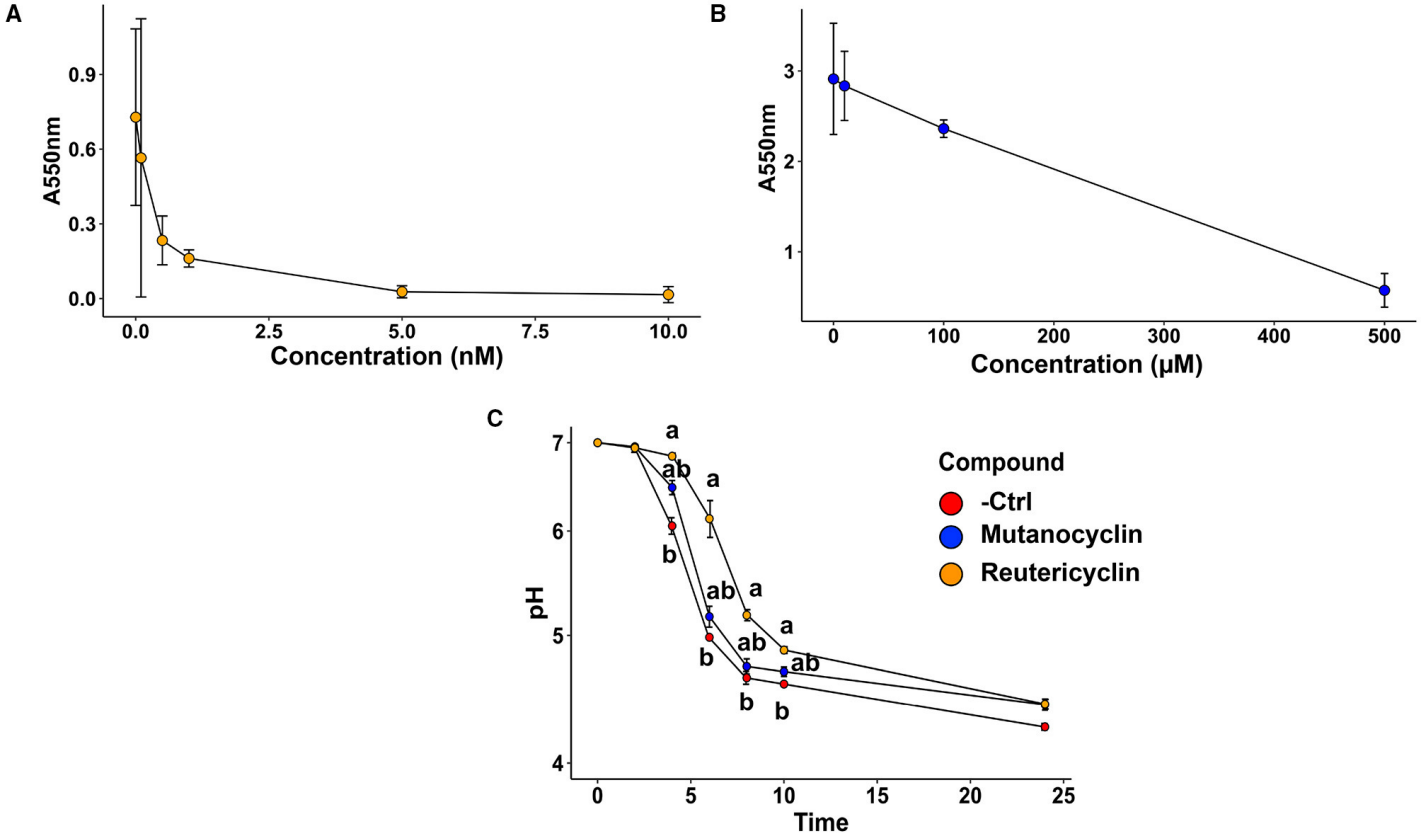
The effect of reutericyclin and mutanocyclin on biofilm formation and acid production on an *in vitro* oral community. **(A,B)** Crystal violet biofilm assay results showing the dose responses of 0–10 nM of reutericyclin **(A)** and 0–500 μM mutanocyclin **(B)** on salivary biofilm formation after 16 h. **(C)** Effects of 0.5 nM reutericyclin and 500 μM mutanocyclin on the pH of the oral microbial community as a function of time. Statistical significance was determined using the Kruskal-Wallis one-way ANOVA, with a *post-hoc* Dunn's test. Error bars represent SEM.

*The impact of reutericyclin A and mutanocyclin on oral microbiota biofilm formation and acid production*: To assess to what extent reutericyclin A and mutanocyclin affected biofilm formation and acid production, the two main virulence factors of caries-associated biofilms, an *in vitro* oral biofilm model system was employed as described previously and in the Section “Materials and methods” [20, 21]. Briefly, saliva was collected from a healthy donor and used to inoculate SHI media. The resulting overnight anaerobic incubation was used to make glycerol stocks for downstream experiments. Biofilms were grown using the glycerol stocks to inoculate brain heart infusion (BHI) + 22 mM sucrose in 24-well plates using a pellicle of dried and UV-sterilized saliva. During inoculation, the biofilm community was amended with 0, 0.1, 0.5, 1, 5, or 10 nM reutericyclin or 0, 10, 100, or 500 μM mutanocyclin. Purified reutericyclin was purchased from MedChemExpress (Monmouth Junction, NJ, USA), and mutanocyclin was synthesized from D-leucine ethyl ester hydrochloride and 2, 2, 6-trimethyl-4H-1, 3-dioxin-4-one and purified as described previously [16]. Following 16 h of incubation, a crystal violet biofilm staining showed a logarithmic decrease in biofilm formation in the presence of increasing concentrations of reutericyclin, with a 50% decrease in biofilm formation that occurred with < 0.5 nM (Figure 2A). Mutanocyclin was much less effective at inhibiting biofilm formation, with increasing concentrations of mutanocyclin producing a response with a 50% decrease in biofilm formation that did not occur until the range of 100 to 500 μM (Figure 2B). Based on these results, the concentration chosen for subsequent experimental procedures on the effect of the compounds was 0.5 nM for reutericyclin and 500 μM for mutanocyclin, a 1,000,000-fold difference in concentration between the two, which would significantly inhibit the biofilm community while still allowing for some growth. These concentrations were used in downstream biofilm experiments to measure effects on acid production (Figure 2C) and community composition (16S sequencing). For measurements of acid production, the pH of the supernatant was monitored every 2 h after compound amendment. 0.5 nM reutericyclin significantly inhibited acid production at the 4-, 6-, 8-, and 10-h time points (Figure 2C). 500 μM mutanocyclin slightly inhibited the acid production of the community, but the difference from the control was not statistically significant (Figure 2C). Taken together, these results suggest that reutericyclin might significantly impair the cariogenic potential of a dental plaque biofilm, while mutanocyclin had little effect.

**Figure 3:**
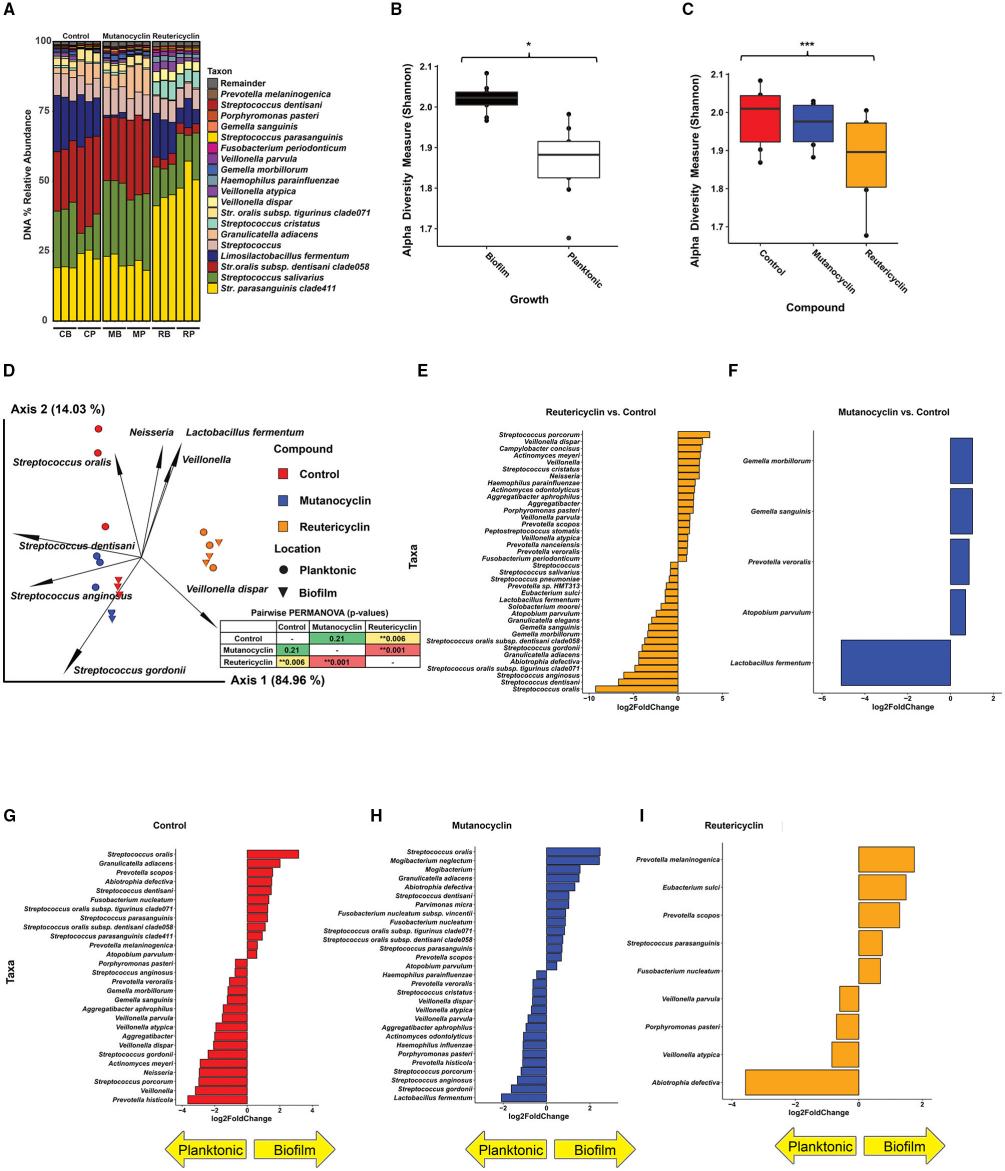
The effect of reutericyclin and mutanocyclin on an *in vitro* oral community. **(A)** Stacked bar plots of 16S rRNA gene (16S) sequencing analysis showing percent abundances of taxa across all conditions tested. C = control, M = mutanocyclin, R = reutericyclin, B = biofilm, P = planktonic. **(B,C)** The effect of reutericyclin and mutanocyclin on alpha diversity in an oral microbial community. Alpha diversity (Shannon metric) of 16S sequencing grouped by A: Growth phase and B: Compound. Statistical differences between groups was assessed using Kruskal-Wallis test. ^*^highlights *p* < 0.05 and ^***^highlights *p* < 0.001. **(D)** PCA biplot showing beta diversity (Robust Aitchison metric) and feature leadings generated with the QIIME2 [22] DEICODE plugin [23]. Data points represent the indicated samples and vectors illustrate the feature loadings (i.e. taxa) driving the differences in ordination space. Statistical differences between replicates assessed using a pairwise PERMANOVA analysis, displayed in the inset. ^**^highlights *p* < 0.01. **(E,F)** Taxa with significantly different abundances upon treatment with reutericyclin or mutanocyclin. Bar charts illustrating the log2 fold change of taxa that had false discovery rate (FDR)-corrected adjusted *p*-values < 0.05 (based on DESeq2) upon treatment with either 0.5 nM reutericyclin **(E)** or 500 μM mutanocyclin **(F)**. **(G–I)** Taxa found in the biofilm versus the supernatant. Bar charts illustrating the log2 fold change of taxa that had false discovery rate (FDR)-corrected adjusted *p*-values < 0.05 (based on DESeq2) in the biofilm or in the supernatant (planktonic) in the negative control **(G)**, or upon treatment with either 0.5 nM reutericyclin (H) or 500 μM mutanocyclin **(I)**.

*Amendments with reutericyclin and mutanocyclin impact the relative abundance of taxa present in the in vitro oral biofilm community*: To examine the effects of reutericyclin and mutanocyclin on the taxonomic profile of the oral biofilm community, 0.5 nM reutericyclin in 20% dimethylsulfoxide (DMSO), 500 μM mutanocyclin in 20% DMSO, or 20% DMSO (control) were added to replicate wells (three replicate growth wells per compound and control). After 16 h of growth, the communities were examined by 16S sequencing (Supplementary Table 1). Overall, *Streptococcus* were the most abundant genera, with *Streptococcus parasanguinis* clade 411 being the most abundant species, followed by *Streptococcus salivarius, Streptococcus oralis* subsp. *dentisani* clade 058, and *Limosilactobacillus fermentum*. Levels of *L. fermentum* appeared to be lower in mutanocyclin-amended communities as compared to the other two conditions (Figure 3A). The alpha diversity of the bacteria in the *in vitro* biofilms was greater than that of planktonic bacteria in the supernatant (Figure 3B). The alpha diversity of the communities amended with reutericyclin was significantly lower than that of the control, while the alpha diversity of the communities amended with mutanocyclin was trended lower than the control but was not significantly different, statistically (Figure 3C). Beta diversity was assessed using a robust Aitchison principal component analysis (PCA) plot [23] (Figure 3D). The results indicated that reutericyclin clearly affects beta diversity, with the taxonomic composition grouping according to the addition of mutanocyclin or reutericyclin. Reutericyclin-amended biofilms showed a more differentiated group as compared to the control and the mutanocyclin-amended groups (see the distribution of 16S sequencing libraries along the first ordination axis in the PCA plot). The biofilm (compared to planktonic communities) was more differentiated in the negative control as compared to the compound-amended groups, with little to no differentiation between the biofilm and planktonic growth with reutericyclin (Figure 3D). Differential abundance analysis of the 16S data using DESeq2 [24] revealed many statistically significant differences in relative abundances of taxa in response to reutericyclin (Figure 3E). Some of the taxa with the largest changes in relative abundance included an increase in *S. oralis, Streptococcus dentisani*, and *Streptococcus anginosus*, and a decrease in *Veillonella dispar, Streptococcus porcorum*, and *Streptococcus cristatus* (Figure 3E). Relative abundance values of *Gemella sanguinis, Gemella morbillorum, Prevotella veroralis*, and *Atopobium parvulum* (all gram-positive bacteria) increased with mutanocyclin (Figure 3F). The effects of 500 μM mutanocyclin on the salivary microbiome compared to the control showed less bacteria were affected overall compared to 0.5 nm reutericyclin treatment.

Conversely, the same taxa decreased significantly in the experiment amended with 0.5 nM reutericyclin. Interestingly, although both mutanocyclin and reutericyclin decreased the levels of *L. fermentum* (gram-positive), mutanocyclin decreased the levels of *L. fermentum* to a much greater extent, and it was by far the taxa most affected by mutanocyclin treatment (Figures 3E,F).

Amendment with the compounds also affected whether particular taxa were more abundant in the biofilm vs. the supernatant (Figures 3G–I). For example, *Abiotrophia defectiva* was more abundant in the supernatant compared to the biofilm during reutericyclin treatment, whereas in both the control and mutanocyclin groups, *A. defectiva* was more abundant in the biofilm. Since reutericyclin treatment decreased *A. defectiva* overall, this may indicate that the dead cells are floating in the media, rather than alive in the biofilm.

**Figure 4:**
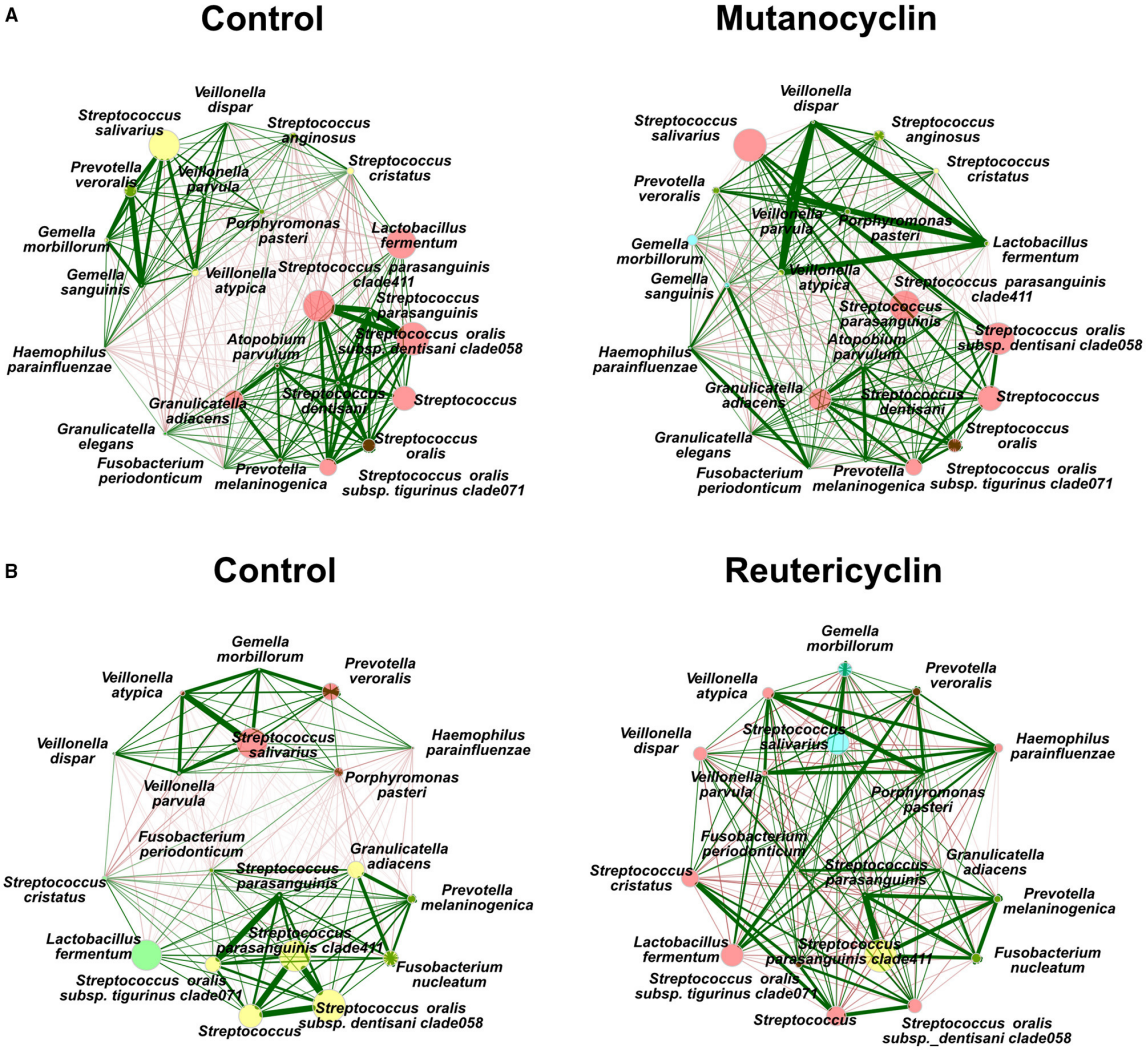
Reutericyclin and mutanocyclin affect microbial correlations in an oral microbial community. Spearman association networks of 16S sequencing of the oral microbial community treated with either 500 μM mutanocyclin **(A)** or 0.5 nM reutericyclin **(B)**, compared to a negative control. Nodes represent the species with the highest variances. Node size represents counts, node color represents hierarchical clustering, red and green edges represent negative and positive associations, respectively, with edge thickness representing higher Spearman's ρ.

To examine the possible changes in co-occurrence and community ecology upon the treatment with the compounds, a network analysis was performed (Figure 4). In the control samples, there were two fairly well-defined groups of correlated taxa; one included *S. salivarius, Veillonella* spp., and *Gemella* spp., and another contained *L. fermentum, S. parasanguinis, S. oralis*, and *Granulicatella* spp. These co-occurring groups were significantly disrupted by the treatment with either compounds. Mutanocyclin treatment caused *L. fermentum* to correlate strongly with *Veillonella* spp. In reutericyclin, *L. fermentum* was correlated to *P. veroralis* and *Haemophilus parainfluenzae*. Collectively, this data indicate that both compounds have an impact on taxonomic diversity and interaction networks between the community members. While reutericyclin had sweeping effects on many taxa, mutanocyclin had a much more subtle and specific effect on reducing the abundance of *L. fermentum*.

*Impacts of S. mutans B04Sm5 on the oral biofilm community*: While the addition of the actual reutericyclin and mutanocyclin compounds did have an effect on the *in vitro* biofilm community to varying degrees, the impact of the producer strain *S. mutans* B04Sm5 carrying *muc* was unknown. To examine this, B04Sm5 or a *muc* mutant, Δ*mucD* (unable to produce either compounds) or Δ*mucF* (capable of producing reutericyclin only) [16], were introduced to the community in separate experiments. Wild-type B04Sm5 produces both mutanocyclin and reutericyclin; however, mutanocyclin is thought to be the “final” end-product, and relatively small amounts of reutericyclin are made [16]. As an additional control, we also introduced *S. mutans* UA159, a well-characterized cariogenic strain, which did not encode *muc*, and therefore did not produce either compound [25].

A pilot experiment was performed to determine the optimal amount of *S. mutans* to be added to the community without causing a community crash (total elimination of other community members). The goal was to maintain measurable amounts of *S. mutans* as well as other oral species after 16 h of biofilm growth. Mid-log phase B04Sm5 was added to the oral microbial community, which had been grown overnight, at ratios of 1:1, 1:10, 1:100, 1:1000, and a negative control with just the community in BHI. 16S sequencing showed that after 16 h, the 1:1 addition of *S. mutans* yielded a community with a 19% relative abundance of *S. mutans*, while the 1:10 yielded a community with a 1.2% relative abundance of *S. mutans*. The lower dilutions and negative control all yielded communities, which had <0.02% relative abundance of *S. mutans*. Therefore, downstream experiments were performed with 1:2 and 1:6 dilutions of *S. mutans*, to yield communities with an *S. mutans* abundance of ~2–10%.

**Figure 5:**
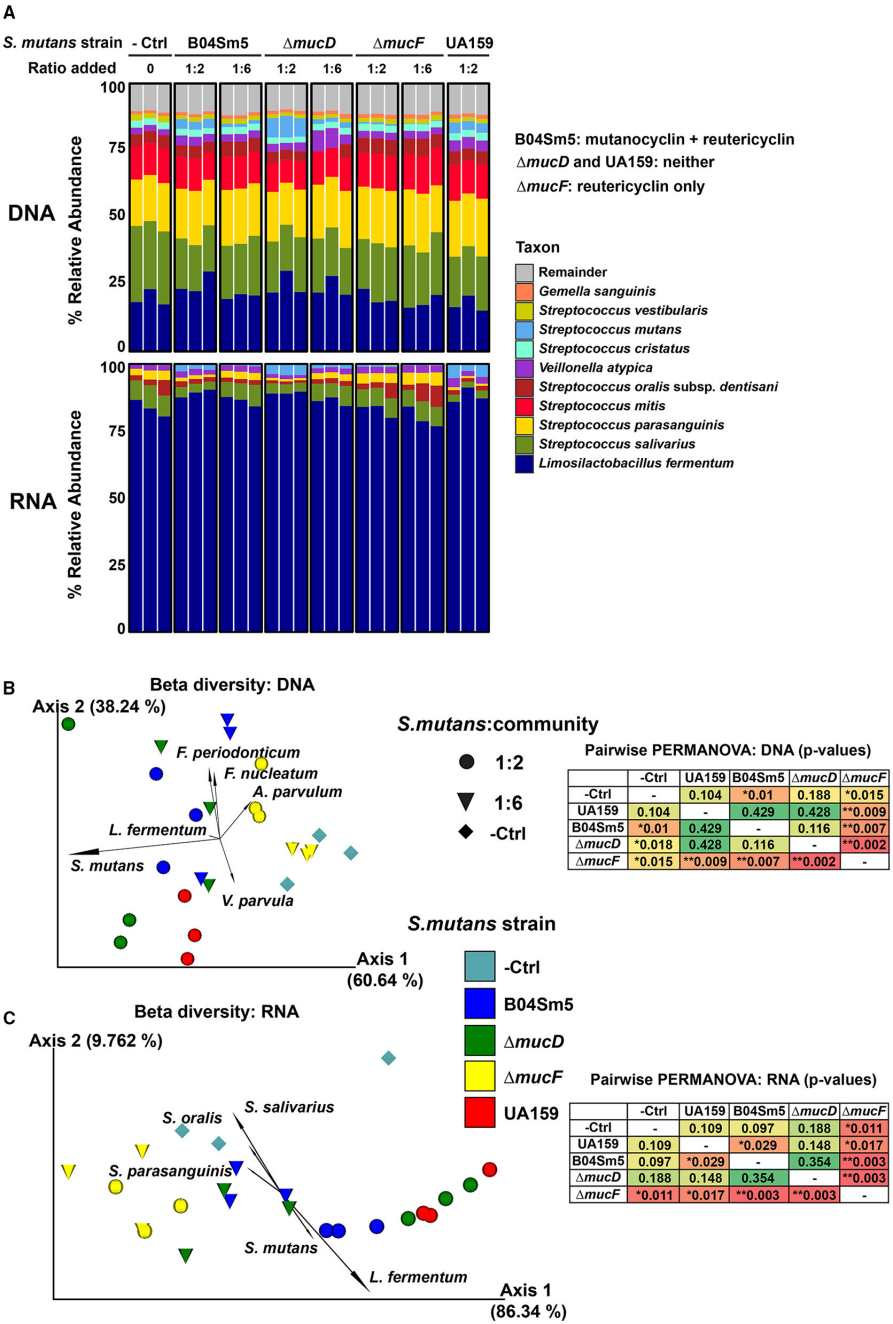
Addition of reutericyclin and mutanocyclin-producing *S. mutans* to an *in vitro* oral biofilm community. **(A)** Stacked bar plots illustrating the % relative abundance of the indicated taxa across the samples with the indicated *S. mutans* strains added at the indicated ratio to the *in vitro* oral community. The results of 16S are shown in the top set of bars (DNA-based), while mRNA sequencing results (based on MetaPhlAn3) are shown in the bottom set of bars. **(B,C)** PCA biplots showing beta diversity [using Robust Aitchison metric in **(B)** and Bray-Cutris in **(C)**]. Data points represent the indicated samples, and vectors illustrate the feature loadings (i.e., taxa) driving the differences in ordination space. The results of 16S sequencing are shown **(B)**, while mRNA sequencing results (based on MetaPhlAn3) are shown **(C)**. Statistical differences between the replicates assessed using a pairwise PERMANOVA analysis, displayed in the insets. ^*^p < 0.05 and ^**^p < 0.01.

Dilutions of B04Sm5, UA159, Δ*mucD*, and Δ*mucF* were added to the oral microbial community in separate replicate growth wells (three replicates per strain). To ensure that the amended dilutions performed as expected, the *in vitro* biofilm communities were examined with 16S sequencing (Supplementary Table 2), which revealed relative abundances ranging from ~0.2% in the 1:6 dilution of Δ*mucF* to 7% in the 1:2 dilution of Δ*mucD*. No *S. mutans* was detected in the negative control samples. The relative abundance of *S. mutans* was reflective of the amounts of cells added (i.e., the dilution) and the growth rate of each strain (Figure 5A). It was shown previously that the growth rates of the sains were Δ*mucD* = UA159 > B04Sm5 >> Δ*mucF*, likely due to the toxicity of reutericyclin [16]. At the DNA level, the increase in *S. mutans* relative abundance seemed to be at the expense of other taxa fairly equally (i.e. no specific taxa had an overrepresented or statistically significant decrease).

To further examine the living and actively transcribing taxa in these communities as well as the expression of particular genes and pathways, RNA-sequencing (RNA-seq) was performed on the same samples. *L. fermentum* was by far the most abundant taxa, based on RNA, with over 80% of all RNA reads coming from the taxa (Figure 5A). As with the DNA-based taxonomic analysis, *S. mutans* abundance reflected both how many cells were inoculated into the community and the growth rate of the strain added (Figure 5A). At the RNA-level, *S. mutans* and *Veillonella infantium* were the only species that showed a significant change in abundance between the groups (based on DESeq2), with both increasing upon the addition of *S. mutans*, regardless of the strain amended. This was contrary to the hypothesis that mutanocyclin-producing *S. mutans* would decrease the abundance of *L. fermentum*, as was seen when purified mutanocyclin was added. Overall, the differing growth rates of the *S. mutans* strains made it difficult to observe whether the carriage of an intact *muc* BGC had any effect on the community. Any effect of mutanocyclin or reutericyclin seemed to be masked by the differing growth rates of the strains. UA159 and Δ*mucD*, both of which do not produce the compounds, were closer to each other in ordination space, than they were to B04Sm5, but again this could be due to a higher growth rate of these strains resulting in higher abundances compared to B04Sm5 and not necessarily anything to do with the production of mutanocyclin or reutericyclin. The differences in growth rates between the strains does indicate that toxic reutericyclin is still being produced by B04Sm5 and Δ*mucF. L. fermentum* did not appear to be inhibited by the addition of mutanocyclin-producing B04Sm5. HumanN3 was performed on the mRNA sequencing data to examine the functional pathways present in the community (Supplementary Tables 4–6). Similar to the taxonomic analysis, the functional analyses of mRNA reads showed no major differences in the gene family or pathway expression between mutanocyclin- or reutericyclin-amended communities. Although many genes in the metatranscriptomic data set were differentially expressed, most were from *S. mutans* and likely to be different between the strains simply because the amount of *S. mutans* cells was different.

To determine whether *muc* was being expressed by the *S. mutans* strains amended to the *in vitro* community, the metagenomic RNA-seq reads were mapped to the B04Sm5 genome (Supplementary Table 7). *muc* was indeed expressed by the B04Sm5, Δ*mucD*, and Δ*mucF* amended to the communities, and was not found in the negative control or communities amended with UA159. Differential abundance testing was performed using DESeq2, and demonstrated that the expression of *mucA, mucB*, and *mucH* was increased, while the rest of the BGC had a decreased expression in the communities amended with Δ*mucD* compared to the communities amended with B04Sm5 (except *mucC*, which was not differentially expressed; Table 1). In the case of the communities amended with Δ*mucF, mucA* had an increased expression, *mucH* was unchanged, and the rest of the BGC had a decreased expression compared to the communities amended with B04Sm5 (Table 1).

**Table 1:** Expression of *muc* genes in Δ*mucD* and Δ*mucF* strains compared to B04Sm5 (log2 fold-change).

**Name**	**Putative product (NCBI-PGAP)**	**ΔmucD**	**ΔmucF**	**Geneid**
*mucA*	3-oxoacyl-ACP synthase	2.58564569	1.19925521	IBL27_RS09110
*mucB*	thiolase family protein	1.32475972	−1.7582417	IBL27_RS09105
*mucC*	2,4-diacetylphloroglucinol biosynthesis protein		−2.3188811	IBL27_RS09100
*mucD*	non-ribosomal peptide synthetase	−14.06934	−1.5771313	IBL27_RS09095
*mucE*	polyketide synthase	−3.5433655	−2.1506834	IBL27_RS09090
*mucF*	HXXEE domain-containing protein	−2.7841312	−5.865363	IBL27_RS09085
*mucG*	TetR/AcrR family transcriptional regulator	−3.9166794	−3.1611157	IBL27_RS09080
*mucH*	TetR/AcrR family transcriptional regulator	1.06763813		IBL27_RS09075
*mucI*	DHA2 family efflux MFS transporter permease subunit	−3.2679608	−3.2504807	IBL27_RS09070
*mucJ*	small multidrug export protein	−2.5618472	−3.3842254	IBL27_RS09065

## Discussion

Although biofilm formation, acid tolerance, and acid production are clearly virulence traits of caries-causing bacteria and dysbiotic dental plaque communities, the intricate ecological, metabolic, and signaling relationships within the complex microbial community are not well understood [1]. *S. mutans* exemplifies the three pathogenic traits described above and is also known to use bacteriocins (called mutacins in *S. mutans*), as well as other small molecules, in its competition with health-associated commensals [8, 26–28]. A subset of *S. mutans* strains encode the *muc* BGC, which produces reutericyclins A-C and mutanocyclin [14, 16]. Reutericyclin A was originally discovered in *L. reuteri*, and is known to have a strong antimicrobial activity against Gram-positive bacteria *via* proton ionophore activity [13, 17, 19]. Mutanocyclin has not displayed antimicrobial activity in previous studies but showed anti-inflammatory properties in a mouse model [15, 18]. *S. mutans* B04Sm5 uses *muc* to produce both mutanocyclin and reutericyclins, although mutanocyclin is produced in larger amounts [16]. The deletion of the *mucD* gene eliminated the production of both compounds, while the deletion of the *mucF* acylase caused the production of reutericyclin only (Figure 1) [16]. *S. mutans* utilized reutericyclin to inhibit the growth of neighboring commensal streptococci [16]. Ecological effects of reutericyclin A (referred to throughout this study as just “reutericyclin”) and mutanocyclin on a broader oral microbiome have not yet been described. Here, by using an *in vitro* oral microbial biofilm model, changes in community composition and function were assessed at the gene and pathway level following additions of the *S. mutans* strains that produce reutericyclins and/or mutanocyclin.

Purified reutericyclin potently inhibited biofilm formation by the oral community, while mutanocyclin only inhibited biofilm formation at concentrations 200,000–1,000,000-fold higher than that of reutericyclin (Figure 2). Because an effect of mutanocyclin was observed at 500 μM, and an effect of reutericyclin was observed at 0.5 nM, subsequent experiments were performed at these concentrations. Furthermore, reutericyclin was able to inhibit acid production of the oral community (Figure 2). Because reutericyclin is also bactericidal to many members of the community, it is not yet clear whether there is additional activity that specifically inhibited biofilm formation and acid production, or whether the observed decreases were simply the result of cells being killed by reutericyclin. The higher alpha diversity of the biofilm community compared to the planktonic community is likely due to the stratified structure creating metabolic gradients and opening more diverse ecological and biogeographical niches.

The addition of either reutericyclin or mutanocyclin to the biofilm community resulted in significant changes in relative abundances of several taxa in the community (Figure 3). Reutericyclin significantly altered the abundances of nearly 40 taxa. Despite its reported antimicrobial spectrum of activity being broad against Gram-positive bacteria [18], various Gram-positive bacteria were both among the most significantly increased and decreased with reutericyclin treatment. It is not clear whether reutericyclin is actually less damaging to the species that increased in abundance, or whether those species are protected through another mechanism, such as being in less penetrable areas of the biofilm. Mutanocyclin caused significant effects on the abundances of only five taxa, although they were modest increases with the exception of a large decrease in *L. fermentum*. Further research is in progress examining whether *L. fermentum* is specifically inhibited by mutanocyclin, or whether this decrease is due to an indirect mechanism. It is worth noting that the effects of mutanocyclin occur at 500 μM, which is a very high concentration that may not be physiologically relevant *vis-à-vis* what *S. mutans* normally produces in a multispecies biofilm. *In vitro* and *in vivo* concentrations of reutericyclin and mutanocyclin produced by *S. mutans* within a biofilm community are not known. Another limitation of the *in vitro* oral biofilm community is that some species may survive the freezing and thawing better than others, skewing the taxonomic composition compared to the original saliva. Additionally, the saliva from only one donor was used to seed the *in vitro* communities used here, therefore the composition and effects of reutericyclin and mutanocyclin might be different when oral microbiotas from other donors were examined. The correlation network analysis does suggest that the addition of either compounds may change the ecological stratum of the community, and which taxa have been found in the biofilm vs. planktonically (Figure 4). Interpretation of the changes in taxa found in the biofilm vs. the supernatant is limited by the lack of differentiation between living and dead cells.

Because the purified reutericyclin and mutanocyclin affected the *in vitro* oral biofilm community, the effect on the community of *S. mutans* producing these compounds was also tested. As described earlier, B04Sm5 produces reutericyclins A-C and mutanocyclin, while Δ*mucD* and UA159 do not produce any of these compounds, and Δ*mucF* only produces reutericyclins [16]. The effort to incorporate *S. mutans* into the biofilm community and have it persist as a metabolically active member for 16 hours was successful. The optimization of how much *S. mutans* to incorporate in the community has applications beyond this study. As *S. mutans* is not an early colonizer of the tooth surface, to cause disease, it must be able to join and persist in already established biofilms. The protocol developed here can be used for other *S. mutans* strains or other species to examine their impact on the taxonomy and physiology of an *in vitro* oral community. The differing growth rates of the *S. mutans* strains are a confounding factor, and are likely due to the toxicity of reutericyclin. As UA159 and Δ*mucD* do not produce reutericyclin, they grow more rapidly than B04Sm5, which produces small amounts of reutericyclin (of which most is converted to mutanocyclin by MucF) [16]. *mucF* displays severely inhibited growth due to a toxic buildup of reutericyclin, but is also the best at inhibiting neighboring commensal bacteria [16]. The amount of *S. mutans* detected in the communities matched both the amount of *S. mutans* added, and the growth rate of the particular strain, in both the DNA and RNA-seq analysis (Figure 5). The distinct growth rates made it difficult to interpret whether the carriage of *muc* significantly impacted the community. For example, any effect of the inhibition of *L. fermentum* by mutanocyclin, produced by B04Sm5, was too insignificant to observe as *L. fermentum* did not decrease upon the addition of B04Sm5. Of the taxa in this *in vitro* community, *Lactobacilli* are the most aciduric, while *S. mutans* and *Veillonella* are the next most aciduric [29, 30]. This helps explain why after 16 h *L. fermentum* was by far the most active taxa, based on the mRNA sequencing. This time point likely represents a late stationary phase, where all the carbohydrates in the media have been fermented to acid, and only the most aciduric taxa (*L. fermentum*, mainly, but also *S. mutans, Veillonella*, and to a lesser extent *S. salivarius*) are able to continue to perform glycolysis and remain active. It is possible that the addition of *S. mutans* helped the community to “transition” to a more acidophilic state that was more amenable to *L. fermentum* and *V. infantium*, which would make sense given what is known about the caries-associated dysbiosis process [1]. As the taxonomic analysis was only performed at 16 h following inoculation, it is possible that there were effects of reutericyclin and/or mutanocyclin that occurred at earlier time points and therefore were not observed, and that the expression of *muc* at earlier time points may have been different. It is also unclear when and to what extent the relative mRNA abundance of *S. mutans* was the highest. Future experiments could examine a time series following the amendment of *S. mutans* to see if the transition state of the community between the strains was different, and should also examine the pH of the communities to observe how it correlates with taxonomic composition. Biogeographic distribution of taxa within the biofilm may also limit the impact of reutericyclin and mutanocyclin on the community. As *S. mutans* cells are known to spatially organize into a rotund structure surrounded by other bacterial species within biofilms [31], it is possible that reutericyclin and mutanocyclin do not diffuse very far into the areas of the biofilm occupied by other species, limiting the impact of the compounds mainly to *S. mutans* cells themselves. Further experiments are needed to determine the biogeography of *S. mutans* within biofilms grown under the conditions tested here. *muc* was indeed expressed by B04Sm5, Δ*mucD*, and Δ*mucF* amended to the communities, showing that the expected compounds are likely produced in this setting. The complexity of the community experiment makes it difficult to interpret whether changes in *muc* BGC expression between B04Sm5 and Δ*mucD* or Δ*mucF* are due to the deletion of genes, or are due to subtle differences in community taxonomy and biogeography. Further research examining control *muc* expression, and the role of the *mucG* and *mucH* regulators, is currently in progress.

Overall, this study illustrates that reutericyclin has wide-ranging inhibitory effects on an *in vitro* oral biofilm, yet its spectrum of activity across taxa and biofilm biogeography is not completely understood. Mutanocyclin had a specific effect of inhibiting *L. fermentum* at very high concentrations; however, the mechanism of action or any other roles of the compound remain to be elucidated. This study validates the use of an *in vitro* biofilm system to test the ability of cariogenic *S. mutans* strains to actively persist in the community and alter its composition. Because the Illumina- and Nanopore-based 16S sequencing protocols employed here target the entire 16S rRNA gene (V1-V9), discrimination between closely related species of *Streptococcus* was obtained, providing a significant advantage to the single-variable region 16S methods employed by many other oral microbiome studies. The addition of *S. mutans* to the *in vitro* biofilm increased the abundance of *L. fermentum* and decreased the abundance of other streptococci. Lastly, further work is needed to determine how *S. mutans* leverages the production of mutanocyclin and reutericyclins in a complex ecological setting and how this impacts the ability of the community to cause caries disease.

## Materials and Methods

*Bacterial strains and in vitro oral microbiota*: *S. mutans* UA159 and B04Sm5 have been described previously [14, 25, 32]. B04Sm5 carries the *muc* BGC, while UA159 does not. As described previously, Δ*mucD* and Δ*mucF* are the derivatives of B04Sm5 with the respective *mucD* or *mucF* gene replaced with a spectinomycin resistance gene [16]. The Δ*mucD* strain does not produce either mutanocyclin or reutericyclin, while the Δ*mucF* strain produces reutericyclin but not mutanocyclin [16]. For the *in vitro* oral community, a protocol derived from Edlund et al. [20, 21] was employed. Briefly, human saliva was collected from a single healthy human donor into a sterile tube. The donor was not being treated for systemic diseases or taking any prescription or non-prescription medication. Nothing was ingested 2 h prior to and during the collection. About 5 ml saliva was collected and centrifuged at 3,000 rpm for 3 min. About 10 μl saliva supernatant was inoculated into 490 μl SHI media in a 12-well plate. The plate was incubated anaerobically at 37°C for 24 h. After incubation, all wells were collected and consolidated into one tube (8 ml total). About 2 ml glycerol were added to the consolidated anaerobic biofilm incubations and mixed well. Aliquots were made (500 μl) and stored at −80°C until used [12].

### Biofilm Assay

Purified reutericyclin was purchased from MedChemExpress, and mutanocyclin was synthesized from D-leucine ethyl ester hydrochloride and 2, 2, 6-trimethyl-4H-1,3-dioxin-4-one and purified as described previously [16]. A 24-well plate was prepared by coating the bottom of each well with 100 μl fresh saliva, and allowed to air-dry in a sterile hood for 40 min, followed by UV light crosslinking for 40 min. Incubations were prepared by mixing 485 μl BHI (Fisher Chemical, Pittsburgh, PA, USA) + 28 mM sucrose pH7, 5 μl salivary microbiome from glycerol stock aliquot prepared previously, and 10 μl of each compound stock containing reutericyclin or mutanocyclin such that final concentrations of 0, 0.25, 0.5, 1, 5, and 10 nM were obtained for reutericyclin, and 0, 10, 100, and 500 μM were obtained for mutanocyclin (i.e., the compounds were added to the medium with inoculum, not to previously formed biofilms). A control consisted of the same, but with 10 μl 20% DMSO without compound. All plates were incubated in an anaerobic chamber at 37°C for 16 h. For crystal violet staining, after incubation, the media were removed from the plates by inverting the plates over a container with bleach. The biofilms were rinsed with water by immersing the plates in DI H_2_O. The plates were air-dried for 1 h upside down to remove all water, after which 250 μl 0.1% crystal violet were was added to each well and incubated at RT 10–15 min. The plates were rinsed with water as previously 3–4 times. Extra water was blotted on paper towels in a sterile hood, and plates were dried upside down overnight. To quantify biofilms, 250 μl 30% acetic acid was added to each well to solubilize the crystal violet-stained biofilm, and incubated 10–15 min at RT. Each sample was mixed by pipetting and transferred to a fresh 48-well plate, and read on a spectrophotometer (Tecan Infinite Nano, Tecan, Zurich, Switzerland) at 550 nm using 30% acetic acid as a blank. Assays were performed in biological triplicate. Based on the data analysis, in which a reduction of, but not a complete elimination of the biofilm was observed, a concentration of 500 μM was chosen for mutanocyclin. For reutericyclin, 0.5 nM was used for the subsequent pH assay and 16s sequencing.

### pH Assay

*In vitro* oral microbial communities were prepared and treated with reutericyclin (0.5 nM) and mutanocyclin (500 μM) as described earlier for the biofilm assay. The pH of the media was measured using a pH probe (Oakton) at the indicated time following inoculation. Assays were performed in biological triplicate.

### 16S rRNA Sequencing of Communities Treated With Reutericyclin or Mutanocyclin (Illumina)

*In vitro* oral microbial communities were prepared and treated with reutericyclin and mutanocyclin as described earlier for the biofilm assay. A 25 μM stock of reutericyclin and a 25 mM stock of mutanocyclin were prepared in 20% DMSO. Three wells for each compound were prepared by adding 485 μl BHI+25mM sucrose, pH 7, followed by 5 μl of a fresh glycerol salivary microbiome stock, and 10 μl of each compound (for a final concentration of 0.5 nM reutericyclin or 500 μM mutanocyclin). These were incubated 16 h in an anaerobic chamber at 37°C. The supernatant and the biofilm were collected separately by carefully transferring the liquid phase to microcentrifuge tubes without disturbing the biofilm. The biofilm was re-suspended in 500 μl phosphate-buffered saline and transferred to fresh microcentrifuge tubes. All samples were stored at −80°C. Genomic DNA was extracted with the Qiagen QIAamp DNA microbiome kit (Qiagen, Germantown, MD, USA). Samples were further purified for sequencing with the Zymo Research Genomic DNA clean and concentrator kit (Zymo Research, Irvine, CA, USA). The Swift Amplicon 16S +ITS Panel (Swift Biosciences, Ann Arbor, MI, USA) was used to sequence the entire V1-V9 16S rRNA from the microbial population. Paired-end sequencing (300bp) was performed on a MiSeq (Illumina, San Diego, CA, USA). For 16S data analysis, fastq files were analyzed with the R-based Dada2 package [33]. Quality filtering of the data consisted in truncating primer sequences and base calls with a quality score <20. Three different 16S rRNA gene sequence databases were used for taxonomic identification, the HOMD database [34], the Silva database [35], and the RDP database [36]. Species-level identifications were called if only one of the databases identified taxa at species-level identifications. Operational taxonomic unit (OTU) tables were created from the planktonic and biofilm phases separately, as well as together to analyze and compare the effects of mutanocyclin and reutericyclin on planktonic vs. biofilm bacteria and the entire summed data set. The 16S data were filtered for feature sums across all samples with counts > 200. Beta diversity using the Robust Aitchison metric and PCA analysis with feature loadings was performed using the DEICODE [23] plugin for QIIME2 [22].

### 16S Sequencing of Oral Microbial Communities With the Addition of *S. mutans* (Nanopore)

For the pilot experiment, a frozen stock of the oral microbial community described earlier was thawed and used to inoculate BHI media, which was grown overnight at 37°C in an anaerobic chamber under 90% N_2_:5%CO_2_:5%H_2_. A frozen stock of *S. mutans* B04Sm5 was used to inoculate BHI media and was grown overnight under the same conditions. The following day, both the community and *S. mutans* were diluted into fresh BHI and grown to mid-log phase, at which point a combination of *S. mutans* and the community were combined, in triplicate, at vol:vol ratios of 0:1, 1:1, 1:10, 1:100, and 1:1,000, and used to inoculate a 24-well plate coated with a salivary pellicle as described earlier. Following 16 h of growth, the biofilm was harvested and 1-ml aliquots were stored at −80°C. DNA was isolated from a 1-ml aliquot using the QIAamp DNA microbiome kit (Qiagen, Germantown, MD, USA) according to the manufacturer's instructions. A full length 16S rRNA library was generated using the 16S Barcoding kit (Oxford Nanopore Technologies, Oxford, UK) and sequencing on a GridION using an R9.4.1 Flongle flow cell (Oxford Nanopore Technologies, Oxford, UK). A downstream analysis was performed using the EPI2ME v3.4.2-6054278 pipeline (Oxford Nanopore Technologies, Oxford, UK). The results of this pilot were used to inform the follow-up experiment, where the goal was to obtain *S. mutans* 16S rRNA relative abundance of 2–10%. The same experimental design and workflow were repeated using the three samples with *S. mutans* community v:v ratios of 1:2 and 1:6 for B04Sm5, Δ*mucD*, and Δ*mucF*, as well as 1:2 ratios of UA159 and a negative control. All mid-log phase *S. mutans* cultures were normalized by dilution to an optical density at 600 nm of 0.1 using a Tecan Infinite Nano. About 0.1 is the approximate OD600 that Δ*mucF* reaches at mid-log (the max OD600 is ~0.2 at the start of stationary phase) due to the impaired growth phenotype of the strain. Only the 1:2 ratio of UA159 was examined because the maximum number of samples on a single nanopore flow cell is 24. This time a full GridION flow cell was used, rather than a Flongle. Read counts per taxa were obtained using EPI2ME. Beta diversity using the Robust Aitchison metric and PCA analysis with feature loadings was performed using the DEICODE plugin for QIIME2. Nanopore sequencing was used here, and Illumina sequencing was used on the 16S analysis earlier because the MiSeq instrument in our sequencing facility was inoperable when this analysis was performed.

### mRNA Sequencing of Oral Microbial Communities With the Addition of *S. mutans*

Total RNA was extracted from 1-ml aliquots of the communities with the addition of *S. mutans*, described in the previous section, using the RNEasy Power microbiome kit (Qiagen, Germantown, MD, USA) as per the manufacturer's instructions. rRNA was depleted using the CRISPRclean Pan Bacterial rRNA Depletion kit (Jumpcode Genomics, San Diego, CA, USA). Short read libraries were prepared using the NEBNext Ultra II Total RNA Library Prep kit (New England Biolabs, Ipswich, MA, USA) and sequenced for 300 cycles on a NovaSeq 6000 S2 (Illumina, San Diego, CA, USA). Integrity of the mRNA and libraries was verified using a Total RNA Nano chip (Agilent, Santa Clara, CA, USA) on a Bioanalyzer (Agilent, Santa Clara, CA, USA). Relative abundances and the predicted read counts of taxa were obtained using MetaPhlAn3 [37]. Beta diversity using the Robust Aitchison metric and PCA analysis with feature loadings was performed using the DEICODE plugin for QIIME2 on the predicted read counts. Functional annotation of the mRNA was performed using HumanN3 [37]. RNA-seq reads were mapped to the B04Sm5 genome using Burrows-Wheeler Aligner (BWA) MEM [38]. The number of reads mapping to each genomic feature (e.g., gene) was determined using the featureCounts tool from the Subread software package [39]. Differential abundance analysis was performed using DESeq2 [24].

### Statistical Analysis

Statistical analysis was done using the R-based (www.r-project.org) packages qiime2R (https://github.com/jbisanz/qiime2R), and the R-based network analysis package NetCoMi [40].

## Data Availability Statement

The datasets presented in this study can be found in online repositories. The names of the repository/repositories and accession number(s) can be found below: NCBI; PRJNA773113.

## Author Contributions

AE conceived the study. CU and JB performed the experiments and analyzed the data. CU, AE, and JB wrote the manuscript. All authors revised, read, and approved the final version of this manuscript.

## Funding

This research was supported by NIH/NIDCR F32-DE026947 (JB), K99-DE029228 (JB), R00-DE024534 (AE), and R21-DE028609 (AE).

## Conflict of Interest

The authors declare that the research was conducted in the absence of any commercial or financial relationships that could be construed as a potential conflict of interest.

## Publisher's Note

All claims expressed in this article are solely those of the authors and do not necessarily represent those of their affiliated organizations, or those of the publisher, the editors and the reviewers. Any product that may be evaluated in this article, or claim that may be made by its manufacturer, is not guaranteed or endorsed by the publisher.
